# Machine learning approaches for the prediction of postoperative complication risk in liver resection patients

**DOI:** 10.1186/s12911-021-01731-3

**Published:** 2021-12-30

**Authors:** Siyu Zeng, Lele Li, Yanjie Hu, Li Luo, Yuanchen Fang

**Affiliations:** 1 Business School, Sichuan University, Chengdu, China; 2 School of Labor and Human Resources, Renmin University of China, Beijing, China; 3 West China School of Nursing, West China Hospital, Sichuan University, Chengdu, China

**Keywords:** Risk prediction, Machine learning, Complication, Cancer of the liver

## Abstract

**Background:**

For liver cancer patients, the occurrence of postoperative complications increases the difficulty of perioperative nursing, prolongs the hospitalization time of patients, and leads to large increases in hospitalization costs. The ability to identify influencing factors and to predict the risk of complications in patients with liver cancer after surgery could assist doctors to make better clinical decisions.

**Objective:**

The aim of the study was to develop a postoperative complication risk prediction model based on machine learning algorithms, which utilizes variables obtained before or during the liver cancer surgery, to predict when complications present with clinical symptoms and the ways of reducing the risk of complications.

**Methods:**

The study subjects were liver cancer patients who had undergone liver resection. There were 175 individuals, and 13 variables were recorded. 70% of the data were used for the training set, and 30% for the test set. The performance of five machine learning models, logistic regression, decision trees-C5.0, decision trees-CART, support vector machines, and random forests, for predicting postoperative complication risk in liver resection patients were compared. The significant influencing factors were selected by combining results of multiple methods, based on which the prediction model of postoperative complications risk was created. The results were analyzed to give suggestions of how to reduce the risk of complications.

**Results:**

Random Forest gave the best performance from the decision curves analysis. The decision tree-C5.0 algorithm had the best performance of the five machine learning algorithms if ACC and AUC were used as evaluation indicators, producing an area under the receiver operating characteristic curve value of 0.91 (95% CI 0.77–1), with an accuracy of 92.45% (95% CI 85–100%), the sensitivity of 87.5%, and specificity of 94.59%. The duration of operation, patient’s BMI, and length of incision were significant influencing factors of postoperative complication risk in liver resection patients.

**Conclusions:**

To reduce the risk of complications, it appears to be important that the patient's BMI should be above 22.96 before the operation, and the duration of the operation should be minimized.

## Background

Liver cancer is the third leading cause of cancer death in the world [1]. Hepatocellular carcinoma (HCC) is mainly composed of liver cancer cells and is the fifth most common cancer worldwide. Surgical resection and liver transplantation are the main methods of radical treatment of liver cancer, and liver resection has been shown to be the most effective approach [2]. Mortality after liver resection has been reduced to 3%, but the incidence of complications after liver resection has remained high [3], occurring in 20–50% of cases [4]. The occurrence of postoperative complications increases the difficulty of perioperative nursing, prolongs the hospitalization time of patients, and leads to large increases in hospitalization costs, and is therefore a serious problem for hepatobiliary surgeons. The incidence of complications is one of the most often-used markers of surgical quality [5]. To improve the quality of liver resection, doctors have put considerable effort into reducing postoperative complications.

Complications of surgical care are a cause of death and disability worldwide. Avoidable surgical complications account for a large proportion of preventable medical injuries and deaths globally [6]. If we can effectively predict the risk of complications, we can take measures in advance to reduce their incidence. The international Study Group for Liver Surgery (ISGLS) has defined the complications after liver surgery, which mainly consist of postoperative hemorrhage [7], liver failure, and bile leakage [7]. The analysis of liver cancer patients prior to liver resection can facilitate the analysis of the causes of complications. This analysis can be used to develop predictive models is established, the identification of to identify high-risk patients [8]. The analysis of complications after surgery can help clinicians in preoperative intervention related indicators, reduce the risk of complications, and indirectly reduce medical costs [9], helping clinicians to provide patients with accurate prognoses and plan for anticipated complications [10]. Liver resection in patients with personalized auxiliary diagnosis and treatment can provide reference data [11].

There has been some research into this issue. Yamanka et.al (1994) did a refinement of a safe limit for hepatectomy prediction scoring system[12].Bo et.al (2015) used nutritional risk index to predict the survival time of liver cancer patient based on cox-regression [13]. Tranchart et al. (2015) using a logistic regression model to identify risk factors for postoperative complications after liver resection [14]. Giustiniano et al. (2020) used a backward multivariable logistic regression analysis to investigate whether a renal resistive index (RRI) could predict complications after hepatic resection [4]. However, those studies used regression analysis, which involves many complex parameters and formulas, as well as the use of a large number of postoperative variables, and the multicollinearity of the variables was not considered. Such predictive methods lack convenience and simplicity, and cannot easily be used for clinical guidance. Also, most doctors can identify characteristics that will lead to complications post-surgery, but it is difficult to know how to prioritize these characteristics.

Machine learning (ML) methods such as deep convolutional neural networks have been used to predict complications and develop predictive models that can capture the occurrence of complications. Kristen et al. [15] created a predictive model for complications at the time of laparoscopic hysterectomy for benign conditions. Bronsert et al. [16] built a ML model that could be used for electronic postoperative complication surveillance. Moghadam et al. [17] used machine learning to produce evidence that there is a correlation between the occurrence of intraoperative or postoperative hypotensive events and various later complications. Ming et al. [18] compared eight different ML methods and found that ML-adaptive boosting had the best performance. Abd El-Salam et al. [19] compared six ML methods to predict complications of liver cirrhosis and found Bayesian Nets to have the best performance.

Such studies have shown that machine learning can significantly improve the accuracy of prediction of disease occurrence and postoperative prognosis. However, few studies have used machine learning to predict the risk of postoperative complications in patients undergoing liver resection. There are literally thousands of machine learning algorithms available, and hundreds more are published each year. The decision as to which algorithm is most suitable for a given problem is daunting [20, 21]. In this study we aimed to identify a machine learning method appropriate for the prediction of liver complications, which was clinically feasible to use.

In this study, we aimed to utilize variables obtained before or during the surgery, to predict when complications present with clinical symptoms. From the 1st of March, 2019 to the 31st of December 2019, we monitored patients undergoing liver resection and strictly controlled the surgical approach. We collected 28 variables, include age, gender, length of stay after surgery and so on. We implemented five machine learning models and identified the most suitable algorithm for predicting the risk of postoperative complications in patients undergoing liver resection. Our second contribution was that, instead of using a large number of variables in the model, we conducted secondary screening of the variables, an approach which reduced the over-fitting of the model, and made the results more clinically informative.

The rest of this paper is organized as follows. In Sect. 2 we introduce our data sources and the five machine learning algorithms we used. In Sect. 3 we present our results, including sample characteristics, compare the prediction performance of the five algorithms, taking into account the importance of the variables and the resulting clinical implications. In Sect. 4 we discuss the results. Section 5 includes concluding remarks and a description of directions for future research.

## Methods

### Data sources

We collected data from 175 patients from West China hospital, Sichuan University. Of these patients, 144 were male and 31 female. We removed information such as name, home address and time of admission. Each instance contains 13 attributes in addition to the output class. According to previous studies of feature selection algorithms used for practical reasons and wide acceptance search, we considered parameters preoperatively and intro-operatively, including demographic parameters, pathological features, and laboratory results to improve the applicability and accuracy of the model in clinical practice [22].

Post-operative complications were experienced by 33% (n = 58) of the patients. Details of the liver resection variable dataset in this experiment are presented in Table 1.

**Table 1:** Attributes of the dataset

NO	Attributes	Type	Description
1	L_complication	Discrete	Occurrence of complications 0: No 1:Yes
2	Resection range	Discrete	Hepatic segments included in liver cancer surgery 1:hepatic segment 1 2: hepatic segment 2 3: hepatic segment 3 4: hepatic segment 4
3	Type of incision	Discrete	Surgical incision shape 1: Surgical incision along the costal margin 2: Surgical incision along the midline of abdomen
4	Length of incision	Continuous	The length of the incision during the surgery
5	BMI	Continuous	Body Mass Index: the ratio of weight in kg to height in meters squared, a standard measure
6	CA	Continuous	Abdominal cavity depth: The distance from umbilicus to vertebral lip
7	Diameter of tumor	Continuous	The diameter of the biggest tumor
8	Number of tumors	Discrete	Count of tumors in the liver
9	Duration	Continuous	Time from the skin incision to definitive abdominal closure
10	Bleeding	Continuous	Volume of bleeding (ml): The amount of bleeding during the surgery
11	Transfusion	Discrete	Whether transfusion was needed 0: No 1: Yes
12	Plasma infusion	Continuous	Volume of plasma infusion (ml) input during surgery
13	Erythrocyte suspension	Continuous	Volume of erythrocyte suspension (ml) during operation

**Logistic regression (LR):** Logistic regression is used when the dependent variable is binary, nominal or sequential, and there are no restrictions for explanatory or independent variables [23]. In non-large-sample epidemiological studies, this approach is widely used as a simple predictor of chronic disease risk [23, 24]. It is possible to predict the probability of belonging to each level of the dependent variable, and also the possibility of directly calculating the odds ratio using the coefficients of the model [23]. To solve the problems of overfitting and multiple contribution lines, we used stepwise regression (TR) to improve the result. The explained variables are used to produce a simple regression for each explanatory variable under consideration, and then the regression equation corresponding to the explanatory variable contributing the most to the explained variable is produced, and the remaining explanatory variables are gradually introduced. After stepwise regression, the explanatory variables retained in the model are both important and not seriously multicollinearity. This approach has been used to study the risk of complications in patients with cirrhosis [25]. As part of the LR algorithm, the performance of TR was also given in the results of Table 4.

**Decision trees:** Decision tree classifiers have been used in a range of clinical studies [26, 27], An important advantage of decision trees is that they do not necessarily require selection of the explanatory variables prior to model building. Moreover, their non-parametric nature allows them to deal with missing values, and they are robust to the presence of outliers [28]. In this paper, two of the decision tree algorithms, C5.0 and Classification and Regression Tree (CART), were implemented.

**C5.0** works by recursively splitting a sample, using the feature that provides the maximum information gain [29]. For each record in the dataset, C5.0 generates predictions and confidence levels.

**CART** is an effective nonparametric classification and regression method. This algorithm constructs a prediction algorithm by building a binary tree.

**Support vector machines (SVMs)**: SVMs are based on the principle of structural risk minimization, in which the training set is mapped into a high-dimensional feature space, using a nonlinear transformation, referred to as the kernel [30]. The SVM is a computational algorithm that can learn from experience and examples to allocate labels to objects [31]. The algorithm has good accuracy even with a limited number of examples [32]. Due to its advantages, SVM is widely used.

**Random forest (RF)**: A random forest is a classifier that combines multiple decision trees, and its output categories are determined by the mode of the categories output by an individual tree. It is a kind of integrated algorithm, and is often used to improve the prediction accuracy and stability of a model [33].

The functions “glm()”, “step()”, “C5.0” in the package of C50, “rpart()” and “prune.part()” in the package of rpart, “svm()” in the package of e1071, “randomForest()” in the package of randomForest were used to implement the above algorithms by R.

### Performance evaluation

The performance of the ML models was evaluated using the Area Under the ROC Curve (AUC). Meanwhile, the accuracy of the prediction was considered too.

Filter, wrapper and embedded methods are the commonly used methods for feature analysis in machine learning. The methods used to rank the importance of factors include variance, correlation coefficient, maximum information gain and so on. For example, LR uses the correlation coefficient to rank the feature importance, while SVM uses information gain to rank the importance. For each algorithm, the five most significant characteristic variables were identified. Then, based on the occurrence frequency and experts’ opinion of early preoperative intervention, three characteristic variables were selected. Using logistic regression, the risk of postoperative complications in patients undergoing liver resection was analyzed and discussed.

## Results

### Sample characteristics

The proportion of patients with post-surgery complications was 33% (n = 58), The proportions of the training set and the test set were 70% (n = 122) and 30% (n = 53) respectively. All of the patients were under 65 years old, with an average age of 49.8. Of them, 144 (82.29%) were male, and 31 (17.71%) were female. Table 2 presents the characteristics of the datasets. To better understand the baseline characteristics of patients, we divided them into two groups, with complication and without complication, as shown in Table 3.

**Table 2:** Patient characteristics (n = 175)

Risk factor	Mean	SD	Min	P_25_	P_50_	P_75_	Max
BMI (kg/m^2^)	22.52	2.96	16.41	20.25	22.49	24.27	32.85
Length of incision (cm)	23.30	4.61	13	19	24	27	35
CA	0.34	0.04	0.2	0.31	0.34	0.37	0.46
Diameter of tumor (cm)	5.69	3.39	1.0	3.0	5.0	6.9	18.0
Duration (min)	201.04	58.89	70	160	195	235	375
Bleeding (ml)	380	307.82	50	200	300	500	1600

**Table 3:** Baseline patient characteristics in the complications group and non-complications group (N = 175)

Variables	Variable category (assignment)	Sample size (N %)	Complications group (N=58)	Non-complications group (N=116)	Kruskal-Wallis chi-squared	P-Value
*Demographic information*						
Age	18–45:1	50 (28.57%)	14 (24.14%)	36 (30.77%)	0.83	0.3621
	45–65:2	125 (71.42%)	44 (75.86%)	81 (69.23%)		
Gender	Male (1)	144 (82.29%)	48 (82.76%)	96 (82.05%)	0.01	0.9084
	Female (2)	31 (19.61%)	10 (17.24%)	21(17.95%)		
BMI (kg/m2)	Continuous variable ($mean\pm SD$)	22.52$\pm$2.96	21.93$\pm$2.44	22.81$\pm 3.16$	3.16	0.0750
Tumor diameter	Continuous variable ($mean\pm SD$)	5.69$\pm 3.40$	6.58$\pm 3.49$	5.26$\pm 3.28$	70.94	0.1577
Tumor number	1 (1)	145 (82.86%)	46 (79.31%)	99 (84.61%)	5.19	0.3935
	2 (2)	16 (9.14%)	4 (6.90%)	12 (10.23%)		
	3 (3)	7 (4.00%)	4 (6.90%)	3 (2.56%)		
	4 (4)	4 (2.29%)	2 (3.45%)	2 (1.70%)		
	5 (5)	2 (1.14%)	1 (1.72%)	1 (0.85%)		
	6 (6)	1 (0.57%)	1 (1.72%)	0 (0%)		
CA	Continuous variable ($mean\pm SD$)	0.34$\pm$0.04	$0.34\pm$0.046	$0.35\pm$0.044	0.33	0.5661
*Surgical information*						
Incision type	Kocher’s incision (1)	129 (73.71%)	51 (87.93%)	78 (66.67%)	9.00	0.0027
	Abdominal incision (2)	46 (26.28%)	7 (12.07%)	39 (33.33%)		
Incision length	Continuous variable ($\mathrm{mean}\pm \mathrm{SD}$)	23.31$\pm 4.61$	24.78$\pm 3.67$	22.58$\pm 4.87$	37.70	0.0494
Duration	Continuous variable ($mean\pm SD$)	201.04$\pm 58$	234.1$\pm 59.47$	184.64$\pm 51.44$	64.60	0.0401
Incision range	One liver segment (1)	37 (21.14%)	5 (8.62%)	32 (27.35%)	16.29	0.0009
	Two liver segments (2)	55 (31.42%)	16 (27.59%)	39 (33.33%)		
	Three liver segments (3)	44 (25.14%)	15 (25.86%)	29 (24.78%)		
	Four liver segments (4)	39 (22.28%)	22 (37.93%)	17 (14.53%)		
Bleeding	Continuous variable ($mean\pm SD$)	380$\pm 307.82$	516.72$\pm 382.87$	312.22$\pm 236.90$	34.27	0.0243
Transfusion	Yes (1)	10(5.71%)	6(3.43%)	4(2.28%)	3.43	0.0630
	No (2)	165(94.28%)	52(29.71)	113(64.67%)		
Erythrocyte suspension	Continuous variable ($mean\pm SD$)	0.12+0.54	0.28$\pm 0.85$	0.042$\pm$0.26	5.05	0.0240
*Outcome variables*						
Complications	No (0)	117 (66.48%)				
	Yes (1)	58 (33.52%)				

**Fig. 1:**
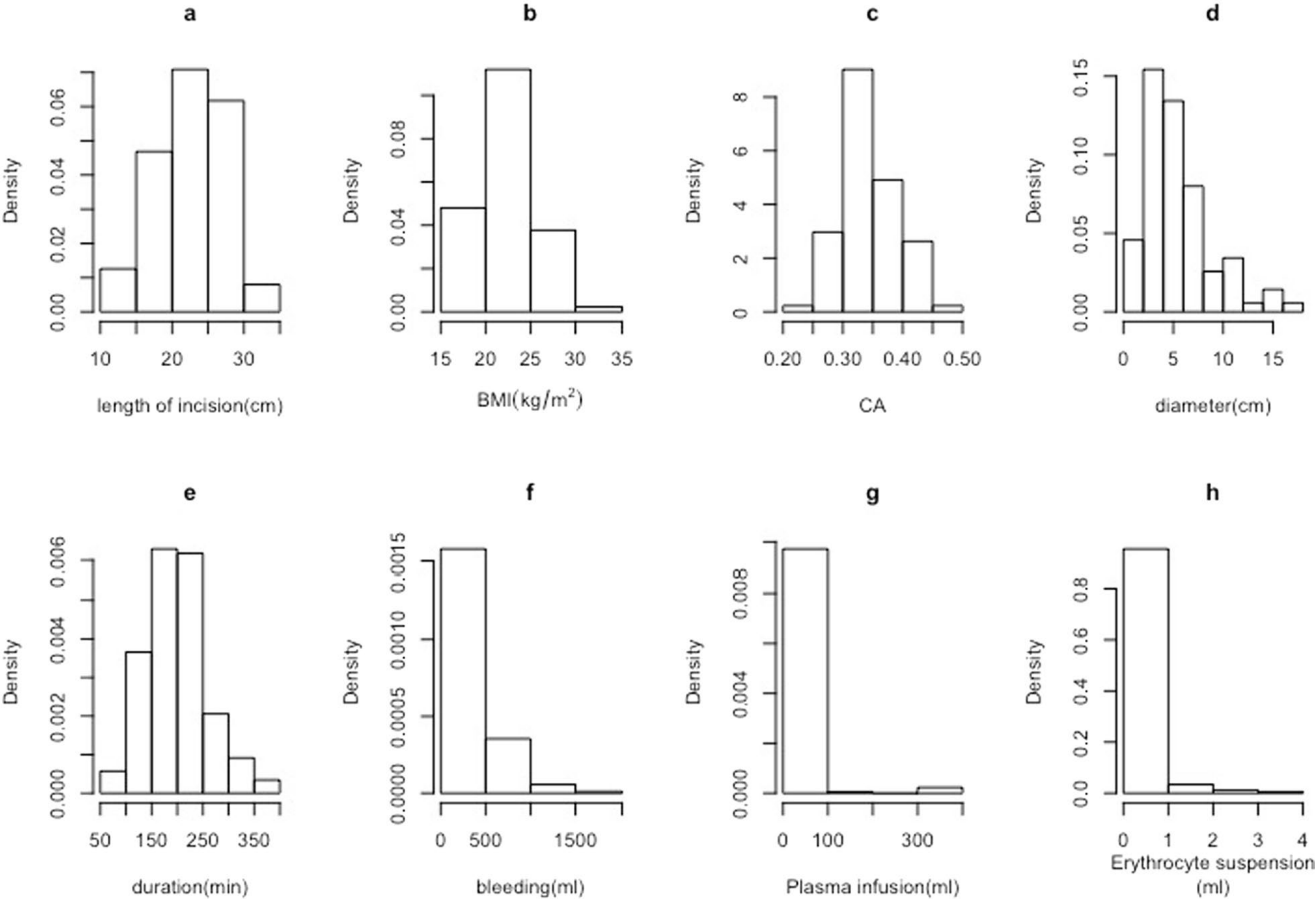
**a** The distribution of length of incision. **b** The distribution of BMI. **c** The distribution of CA. **d** The distribution of duration. **e** The distribution of duration. **f** The distribution of Volume of bleeding during operation. **g** The distribution of Volume of plasma infusion during operation. **h** The distribution of patient Volume of erythrocyte suspension during operation

Figure 1 gives the distribution histogram of the continuous variables. Due to anorexia in patients with liver cancer [34], it is apparent from the BMI figures that most of the patients were underweight. The mean of CA is close to the clinical threshold (0.35). The amount of bleeding during surgery was clinically negligible in most patients.

### Prediction accuracy of different models

**Fig. 2:**
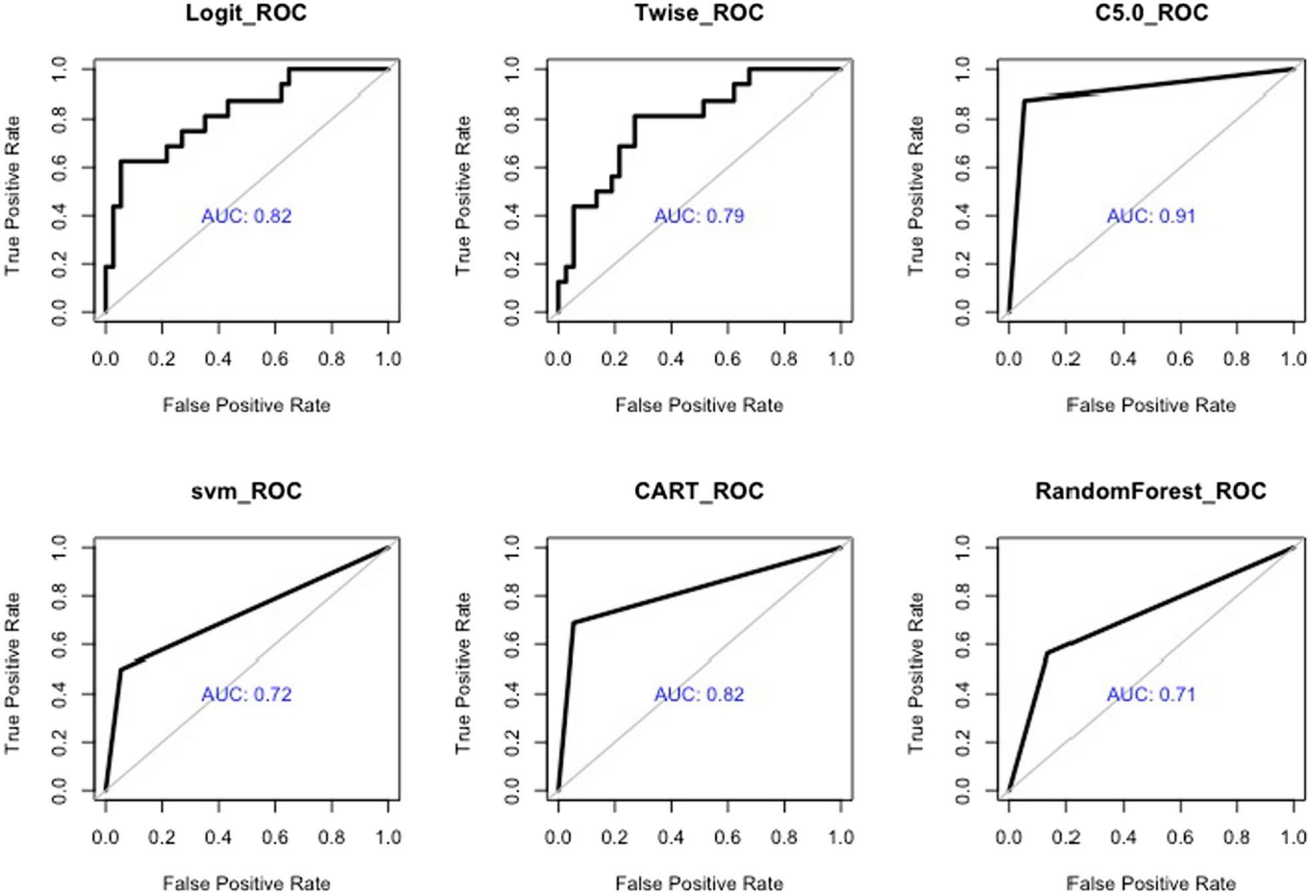
ROC analysis of the different ML algorithms. The AUC for each algorithm is indicated on the diagram. The C5.0 decision tree algorithm is had the best AUC

Figure 2 and Table 4 show the prediction ability for the models. All six machine learning algorithms showed high accuracy in predicting the risk of complications. The accuracy of LR, TR, C5.0, CART, SVM, RF were 0.83 (95% CI 0.77–0.89), 0.79 (95% CI 0.73–0.84), 0.92 (95% CI 0.83–1), 0.87 (95% CI 0.80–0.94), 0.81 (95% CI 0.75–0.87), and 0.77(95% CI 0.71–0.84), respectively. The AUC of the six algorithms was 0.82 (95% CI 0.66–0.98), 0.79 (95% CI 0.66–0.92), 0.91 (95% CI 0.77–1), 0.82 (95% CI 0.70–0.94), 0.72 (95% CI 0.59–0.85), and 0.71 (95% CI 0.60–0.81), respectively. Table 4 shows the sensitivity and specificity of the algorithms. In two sets of regression models, the AIC of the LR model was 196. The TR model was based on the LR. There are only three significant variables left in the TR model after backward and forward improvement based on the LR model. Only two of the 12 variables in LR were significant. Kappa was 19073, which means there is serious multicollinearity in the variables of the LR model, and there may therefore be overfitting of variables in the LR model. The performance of the random forest model was weaker than that of the other models with an AUC value of 0.71(95% CI 0.60–0.81), an accuracy of 77.36% (95% CI 71%-84%), the sensitivity of 56.25%, and specificity of 86.48%. The C5.0 decision tree model had an AUC value of 0.91 (95% CI 0.77–1), with an accuracy of 92.45% (95% CI 83%–1), the sensitivity of 87.5%, and specificity of 94.59% on independent test data (Table 4). This approach significantly outperformed the other models. To provide more accurate clinical advice to doctors, key risk factors were analyzed using C5.0 in the subsequent analysis. We compared our results with those from the literature (Table 5).

**Table 4:** Performance of the different algorithms

Type of ML	Accuracy (95% CI)	Sensitivity	Specificity	AUC (95% CI)
Logistic Regression (LR)	0.83 [0.77,0.89]	0.72	0.60	0.82 [0.66,0.98]
T-wise Regression (TR)	0.79 [0.73,0.84]	0.70	0.59	0.79 [0.66,0.92]
Decision Tree: C5.0 (C5.0)	0.92 [0.83,1]	0.87	0.94	0.91 [0.77,1]
Decision Tree: CART (CART)	0.87 [0.80,0.94]	0.69	0.91	0.82 [0.70,0.94]
Support Vector Machine (SVM)	0.81 [0.75,0.87]	0.50	0.94	0.72 [0.59,0.85]
Random Forest (RF)	0.77 [0.71,0.84]	0.56	0.86	0.7 [0.60,0.81]

**Table 5:** Comparison of different models

No	Authors	Techniques	Diagnosis	Accuracy
1	Our best method	C5.0 decision tree	Liver cancer	0.9245
2	Ming et al. (2019) [18]	ML-adaptive	Breast cancer	0.9017
3	Bronsert et al. (2019) [16]	Binomial generalized linear model	Various diagnosis from electronic health record	0.88
4	Feng et al. (2019) [35]	Logistic regression and Twenty-two machine learning (ML) models	Traumatic brain injuries	0.88
5	Abd El-Salam et al. (2019) [19]	Bayesian Nets	Liver cirrhosis	0.689

### Decision curves analysis of the different models

**Fig. 3:**
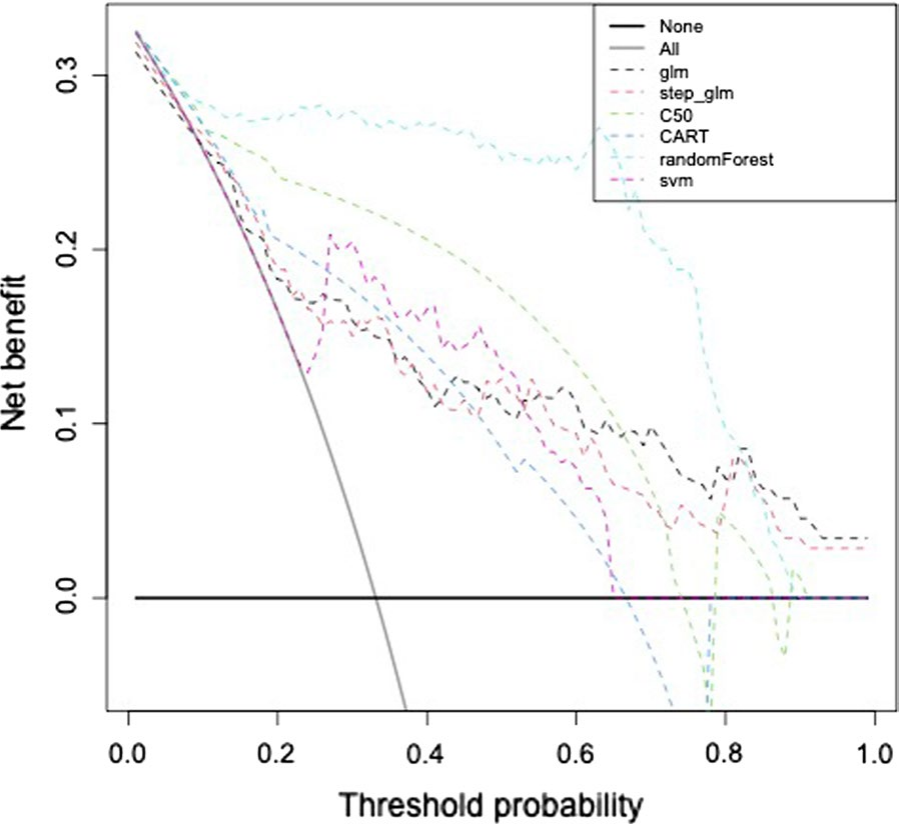
Decision curve analysis of different models for the prediction of postoperative complication risk in liver resection patients

Decision curve analysis is a method to evaluate prediction models and diagnostic tests. It was introduced by Vickers and Elkin in 2006, to overcome the limitations of traditional measures and techniques for evaluating alternative diagnostic and prognostic strategies [36, 37]. Figure 3 shows the decision curve analysis of the models, and random forest shows the best performance.

### Ranking of importance of the variables by different models

Table 5 shows the most influential variables in each of the ML algorithms, and the relative ranks of the top five variables in decreasing order. The importance of each variable was different for each model. Surgical duration was of primary importance in each algorithm. In addition, BMI was an important indicator, and was included in the top five variables of each algorithm. All of the algorithms can be used to provide a threshold for reducing the risk of complications through the control of key risk factors. For example, according to CART, to reduce the risk of complications we need to take into account the following variables: in the low-risk group the diameter of tumor < 6.25 cm, CA < 0.3285, duration < 227.5 min, BMI < 21.755 kg/m^2^, and length of incision < 23.5 cm.

We ranked the risk factors for each algorithm, top five important factors for each algorithm are shown in Table 6, a total of seven important variables, consisting of preoperative variables (BMI, diameter of tumor, length of incision) and intraoperative variables (duration, CA, plasma transfusion, bleeding). Some of these variables, such as tumor size, are hard to control, while others are easy to control to reduce the risk of complications. In this work, we selected three risk factors which can be controlled through reasonable clinical and surgical options. The three variables are duration, BMI, and length of incision.

**Table 6:** Top five risk factors in descending order of importance for the five ML algorithms

Type of ML	First factor	Second factor	Third factor	Fourth factor	Fifth factor
Decision Tree: C5.0	Duration	BMI	CA	Plasma infusion	Length of incision
Decision Tree: CART	Duration	Diameter of tumor	BMI	CA	Length of incision
Support Vector Machine (SVM)	Duration	BMI	CA	Erythrocyte suspension	Length of incision
Random Forest (RF)	Duration	BMI	CA	Diameter of tumor	bleeding

### Clinical guidance for important controllable variables

The three most significant factors—duration, BMI, and length of incision, have been identified by the ML algorithms in the previous sections. Logistic regression, which is the most widely used method in clinical practice [38, 39], can learn and quantify the relations between features and target value. As shown in Table 7, for every unit increase in BMI, the odd ratios of complications decreased by 24 percent; for each additional ten minutes of surgery, the odd ratios of complications increased by 17.49%; and an increase in the length of the surgical opening by one unit increased the odd ratios by 10.54%. The model had values as follows: logit (pi) = −  1.39 + 0.1054 (length of incision) + 0.0174 (duration) − 0.2429 (BMI). To reduce the risk of postoperative complications in liver resection patients, preoperative BMI should be at least 22.96, and the duration of the operation should be limited to 290 min.

**Table 7:** Probability of belonging to each level of the dependent variable

Risk factor	Estimate	Std. Error	z value	Pr ( >|z|)
Length of incision	0.105457	0.047442	2.223	0.026227*
BMI	− 0.242907	0.070834	− 3.429	0.000605**
Duration	0.017495	0.003912	4.472	7.73e−06**

^*^P < 0.05; **P < 0.01

## Discussion

Various risk factors for complications after operation have been described in the literature. In this study, we computed the predictive accuracy of five machine learning models, compared their performance, and ranked the importance of the variables according to the different ML algorithms. C5.0, a decision tree algorithm, had the best performance in predicting the risk of complications after liver resection, with an AUC of 0.91 (95% CI 0.77–1), an accuracy of 92.45% (95% CI 83%-1), a sensitivity of 87.5%, and specificity of 94.59%. The biggest contribution of this paper is that after we conducted a study of the most important risk factors found by the different algorithms, we developed concise and practical clinical suggestions.

Recent literature has reported early complication rates after resection ranging from 20 to 50%. Of our patients, 33% experienced complications. Previous studies tended to focus on only one variable, such as duration of surgery, bleeding, requirement for blood transfusion, and BMI. However, no recent literature has provided clinical guidance based upon these findings.

Machine learning techniques have shown great potential in the field of healthcare management [40], but every model has its limitations. For example, LR uses a large number of variables of low significance to produce accuracy, resulting in over-fitting. Support vector machines are not robust when using large datasets. Due to the differences between algorithms, the ordering of importance of variables is different in different machine learning methods, and the variables selected by a single method may lead to a loss of information. Our study also found that the important variables identified by different methods were different (Table 3).

Most clinicians lack sufficient expertise in machine learning to build a model [40]. Experts in machine learning algorithms, however, generally cannot use models to give clinical advice. We compared the performance of six machine learning algorithms in predicting the risk of postoperative complications in patients undergoing liver resection, and selected the best method based on its prediction accuracy. We then established a secondary learning model, and developed suggestions for issues to be considered before surgery, which has not been done in previous studies. We controlled some risk factors within a certain range, predicted which patients would be at high risk after surgery, and increased nursing care for these patients, to reduce the risk of complications.

The accuracy of the C5.0 model selected in this study was higher than those of previous studies (Table 4). We screened the variables selected as important by various algorithms, and selected three variables for secondary learning using C5.0, producing conclusions with strong clinical significance.

Most studies define overweight individuals as those having a BMI ≥ 25 kg/m^2^, and obesity as BMI ≥ 30 kg/m^2^ [40]. A BMI ≥ 25 has been established as an independent risk factor for complications at the time of liver resection. Most patients (74.36%) with liver cancer suffer from malnutrition, because of digestive symptoms such as loss of appetite and increased energy expenditure and metabolic rate [34]. However, previous studies have focused only on overweight or obesity, which is an independent contributor to postoperative liver failure [41]. Of our study subjects, only 35 had a BMI of more than 25 kg/m^2^, accounting for 20% of the total sample. In the second study of the important variables, we found that when the BMI was lower than 22.96 (58% of the total sample), the risk of complications was higher. This observation suggests that clinicians should attempt to increase patients’ BMI to 22.96 before surgery, to reduce the risk of complications after surgery. Weight loss is common among patients with intraabdominal diseases, and is recognized as a risk factor for postoperative complications. However, the definition of thinness is inconsistent. In this paper we provide a quantitative analysis of patients with liver resection.

Surgical duration is an independent predictor of short-term adverse outcomes, but the association between surgical duration and postoperative complications is unclear [42], especially for liver resection, since there have been no previous studies. We found that for each additional ten minutes of surgery, the risk of complications increased by 17.49%. Using the C5.0 algorithm, we established a division of the duration into three ranges: low risk less than 175 min; moderate risk from 175 to 290 min; and high risk greater than 290 min. Keeping the operation time below 290 min could reduce the risk of complications. For patients with a high risk of complications, more attention should be paid to postoperative care.

There are a few limitations with this study. We only collected data from one hospital, so data collection from more hospitals should be considered. We also need data from patients aged over 65. Data on smoking, drinking and other behaviors of patients with liver cancer are missing. In subsequent experiments, we will collect behavioral data from patients. This study only considered the presence or absence of complications, and we will also consider the grade of complications in future studies.

## Conclusions

We compared five different machine learning algorithms, and showed that C5.0, a decision tree algorithm, had the best accuracy when predicting postoperative complications in patients undergoing liver resection. We also conducted secondary investigation of the important variables identified by the different machine algorithms, and developed suggestions for clinical guidance. We should continue to improve the accuracy of the algorithm, to achieve early prediction of the risk of postoperative complications in liver resection patients.

## Data Availability

The data that support the findings of this study are available from West China Hospital, Sichuan University but restrictions apply to the availability of these data, which were used under license for the current study, and so are not publicly available. Data are however available from the authors upon reasonable request and with permission of West China Hospital, Sichuan University.
